# Examining the Extinction of the Barbary Lion and Its Implications for Felid Conservation

**DOI:** 10.1371/journal.pone.0060174

**Published:** 2013-04-03

**Authors:** Simon A. Black, Amina Fellous, Nobuyuki Yamaguchi, David L. Roberts

**Affiliations:** 1 Durrell Institute of Conservation and Ecology, School of Anthropology and Conservation, University of Kent, Canterbury, Kent, United Kingdom; 2 Agence Nationale pour la Conservation de la Nature, Algiers, Algeria; 3 Department of Biological and Environmental Sciences, University of Qatar, Doha, Qatar; University of Illinois at Urbana-Champaign, United States of America

## Abstract

Estimations of species extinction dates are rarely definitive, yet declarations of extinction or extirpation are important as they define when conservation efforts may cease. Erroneous declarations of extinctions not only destabilize conservation efforts but also corrode local community support. Mismatches in perceptions by the scientific and local communities risk undermining sensitive, but important partnerships. We examine observations relating to the decline and extinction of Barbary lions in North Africa. Whilst the extinction predates the era of the scientific conservation movement, the decline is relatively well documented in historical records. Recently unearthed accounts suggest Barbary lions survived later than previously assumed. We use probabilistic methods to estimate a more recent extinction date for the subspecies. The evidence presented for a much later persistence of lions in North Africa, including generations when sightings were nil, suggests caution when considering felid populations as extinct in the wild. The case raises the possibility that captive animals descended from the Moroccan royal collection are closer contemporaries to wild Barbary lions. Furthermore, our results highlight the vulnerability of very small lion populations and the significance of continued conservation of remnant lion populations in Central and West Africa.

## Introduction

Several statements of extinction relating to felids have been proven unreliable, such as the recent rediscovery of the Barbary leopard [1] and the late persistence of the Caspian tiger, recognized as extinct since the early 1970s, yet later found present in Turkey where a local trade in hunted skins persisted into the 1980s suggesting survival into at least the early 1990s [2]. In both cases local people’s observations remained unknown to science and therefore had no impact on conservation policy.

Wild populations of lion (*Panthera leo*), like other large mammalian carnivores, are suffering severe decline and in Africa have contracted sharply over the past 50 years [3], [4]. Outside East Africa, lion populations are fragmented, with remnants in Central and Western Africa threatened with extinction [5], [6]. The present-day status in sub-Saharan Africa mirrors the situation north of the Sahara a century ago where the once extensive lion distribution from North Africa to India was reduced to fragmented populations by the 20^th^ century [7].

The North African ‘Barbary lion’ or ‘Atlas lion’ occupied the Maghreb, the region isolated from the rest of non-arid Africa by the Sahara [8]. Until the 18^th^ century, Barbary lions ranged from the Atlas Mountains to the Mediterranean [7]. Extensive persecution in the 19^th^ century reduced populations to remnants in Morocco in the west, and Algeria and Tunisia further east, all of which were extirpated during the 20^th^ century [9], [10].

The Barbary lion (*P. l. leo*) is considered distinct from the six commonly defined sub-species in the rest of Africa [11], owing to its geographic separation, morphology and unique montane habitat with cold winters. Although the phylogenetic status of lion populations remains unclear [12], [13], recent morphological and genetic studies consistently differentiate northern lions (India to North Africa) from sub-Saharan African lions [9], [14], [15].

The IUCN’s assessments of species status, threats and extinctions have been established over the past four decades [4], which post-dates the extinction of the Barbary lion, so Red List declarations on the sub-species are based on reviews of literature [11]. The IUCN Wild Cats Status Survey and Action Plan recognized that certain captive lions may be descendants of the Barbary lion, designating the subspecies as ‘extinct in the wild’ [11]. Currently all extant African populations are grouped together as a single taxon, *Panthera leo*, with the species listed as ‘regionally extinct’ across its former northern range [16]. Only the Indian population is recognized as a distinct sub-species, *P. l. persica* [17], whilst North African populations (i.e. the Barbary lion) are mentioned in the context of both the IUCN’s Indian and African lion accounts [4].

In the 16–18^th^ centuries many accounts reported lions in the western Maghreb (northern Morocco) near the Atlantic and Mediterranean coasts [7], [18]. Up to the 1830s lions were still seen in these coastal areas, the Rif mountains and the Marmora forest [19], however records remain sparse throughout the 19^th^ century [20]. By 1880 lions had retreated south of the Bou Regreg and Taza passes [19] into the Atlas Mountains and areas bordering the Sahara, where human populations were largely nomadic. Previous commentators suggested Barbary lions were extirpated sometime between 1920 and 1930 [7], [19], [20]. Later sightings, however, have since been documented with the last in the High Atlas in 1942 [18].

In the eastern Maghreb (Algeria and Tunisia) lions frequented coastal-forested regions, the Tell Atlas and the Aurès mountain ranges. By the late 1800s, Tunisian sightings were confined to localities adjacent to the Algerian provinces of Souk Arras and Tebessa. Although no lions were shot in Tunisia after 1891 [7], rumors of their survival persisted in the 1900s in the Khmir Mountains and near Feriana [21]. The population was probably contiguous across the border, corresponding to home range sizes equivalent to those observed in present-day sub-Saharan Africa and India [22], [23], [24]. In Algeria, lions persisted into the 1890s and hunting accounts, the capture of wild cubs and photographs of tame lions were still widely reported [7], [20]. These observations, alongside interview evidence, suggest that a small population survived in Algeria well into the 20^th^ century [25], long after 1893 when Algeria’s supposedly last lion was shot [7].

Several reviews have considered the history of sightings [7], [19], [20], [26], [27] and the IUCN recognizes that lions persisted in Morocco into the 1940s [17]. Nevertheless these reviews have inadvertently missed, ignored or have lacked access to many local sources and literature accounts. A systematic evaluation of the last sightings in the Maghreb will provide more complete insights into persistence of remnant lion populations and the resilience of large mammalian carnivores to human pressure.

Several menageries in Europe held Barbary lions in medieval times [9] and they were popular exhibits in public zoological gardens in the 1800s [7], [20]. By the early 1900s zoos and circuses in Europe and North America often promoted their lions as “Barbary” [13], although true representatives were said to be only found in the collection of the Sultan of Morocco, derived from animals caught by local tribes [20]. The significance of this collection was not recognized until the 1970s after the lions were moved from the Royal Palace, Rabat, to a new zoo at Temara when a study identified animals with physical characteristics of the Barbary lion [20]. Despite several attempts, a formal scientific breeding program is yet to be established (Frey pers. comm.). Nevertheless, captive breeding has experienced a recent renaissance [28], [29] and a studbook for these animals (hereafter ‘Moroccan Royal lions’) has been developed [13].

Debate surrounds the authenticity of Moroccan Royal lions as descendants of wild Barbary lions [13]. One concern is that Moroccan Royal lions hybridized with sub-Saharan African lions potentially introduced to the collection before the 1970s [20]. Definitive genetic matches have not yet been established between Moroccan Royal lions and wild Barbary lions [13]. The few studies of Barbary lions that utilize museum samples [14] are limited by the scarcity of wild-origin reference specimens. Most recent genetic studies [30], [31], [32] rely on Royal lion samples from two zoos, covering at most 4 (possibly only 2) of the 12 maternal bloodlines [13]. In the absence of genetic data, owing to the lack of verified wild specimens, and working on the precautionary presumption that Barbary lions are taxonomically distinct from other lions, assessment of purity relies on ‘pedigree information’ which at best can be taken from the recently established studbook of lions of known ancestry arising from the Moroccan Royal Collection [13].

The North African-Asian population of lions [14] is only represented by today’s Asiatic lion (*c.*350 wild individuals and *c.*100 zoo captives), so the potential significance of captive Moroccan Royal lions (*c.*90 individuals) is not trivial [13]. In the absence of definitive genetic comparisons we examine the generational separation between lions surveyed in Temara Zoo in 1974 and wild-caught Barbary ancestors. This infers the degree of opportunity for hybridization had non-Barbary individuals bred within the Royal collection, and the potential purity and relevance of the extant captive population.

Our study re-examines the historical decline and extirpation of lions in North Africa. We calculate likely extinction dates for different populations and thus patterns of decline. We also examine new estimates of generation time in the captive population originally derived from wild North African ancestors and thus the likelihood of introgression with sub-Saharan individuals.

## Materials and Methods

### 2.1 Sightings Information

Existing literature was reviewed for accounts of sightings and kills by date and location, including documented interviews in addition to recent interviews by the authors (Tables 1–6). The collation includes sightings, photographs, accounts and recollections since 1839. Observations have been taken from recorded accounts from first-hand sources (written recordings of interviews), from direct interviews of eyewitnesses or a first-hand recollection of an eyewitness account; all sightings in the dataset are therefore from second-hand sources or better.

**Table 1:** The last sightings of lions in the Western Maghreb: Morocco to Western Sahara, 1830s–1940s (Rif Mountains, Anti Atlas, Middle Atlas and High Atlas).

	Location	Season/Year	ObservationType	Solitary(0)/Group (1)	Original source	Ref.
1	Guelaya (the region around Melilla)	Before 1839		0		[19]
2	Cap Spartel-Tanger	Before 1839	Shot, male	0	Maalem Hamed	[51]
3	Mamora forest	1839	Frequent lions	1		[51]
4	Rif region	Summer 1839	Many observed and shot	1	Maalem Hamed	[51]
5	Jebel Kebir (Tanger)	1846	Shot, male	0	Hooker and Ball	[19]
6	Megader pass (at 2300 m)	1864	Camp protected against lion attack		Gerhard Rohlfs	[52]
7	Rif mountains	1895	Last lion killed in the Rif	0	de Planhol, 2004	[53]
8	M’Hamid, south of Zagora (Morocco-Algeria border)	*c.*1900	Observations close to water points	1	Local inhabitants	[18]
9	Djebel Ebrit (Ain Leuh-Timahdit)	*c.*1900	Observations	1		[54]
10	Budaa woods (Azrou)	1901	Observations, frequent lions	1	Marquis of Segonzac	[19]
11	Middle Atlas mountains	1911	Observations	1	Engel	[19]
12	Zaián forests, Beni Mgild (Khenifra)	1911	Lions	1		[52]
13	Oued Ifrane	Winter 1917	Observation, male	0		[54]
14	Azrou vicinity	1920	Observation of a single male	0		[20], [55]
15	Middle Atlas mountains	1922	Shot	0		[56]
16	Atlas Mountains (on the Casablanca-Dakar flight)	1925	Male lion seen from the air	0	Photograph Flandrin, M.	[57]
17	Ouiouane area (Ain Leuh)	1930	Observations of few lions, tracks	1		[58], [20]
18	Toubkal massif (now a NP)	Summer 1930	Observations at 3000 m	1	Local residents	[18]
19	Hassi Aggou and Hassi Tighissit (Assa -TanTan)	1935	Observations at water points	1	Bensalem, M.; Ennah, M.	[18]
20	Hassi Aggou vicinity	1939	Observations of 2 lions	1	Hunter told Monteil 1951	[18]
21	Tizi-n-Tichka pass (Marrakesh- Tadderte)	1942	Shot	0	Minet, J.	[18]

NP = one of several National Parks established across the region since the 1940s.

**Table 2:** Lion sightings in the eastern Maghreb of Algeria and Tunisia 1830–1850 (Ksour Mountains, Saharan Atlas, Tell Atlas, Ouled-Nail, Aurès Mountains).

	Location	Season/Year	Observation Type	Solitary (0)/Group (1)	Original source	Ref.
22	Oued Khalaad, Tunisia	1832	Observation of 16 lions	1	Greenville	[59]
23	Djebel Guezoul (Tiaret)	1836	Shot (100 shot during his life)	1	Mohamed Ben Esnoussi	[60]
24	Souk Tleta (Chelif region)	Winter 1837	Shot, male, female and 2 cubs	1	Agha Djendel	[61]
25	Ain Sbaa (Bou Saada)	1840	Male, attacking a sleeping man	0		[62]
26	Djebel Mezioun	Spring 1840	Observation of female and cubs	1		[63]
27	Oued Tegedemt (South Tiaret)	Summer 1840/42	Observations 2	1	Colonel Scott	[64]
28	El Diss vicinity	1843	Observed female, Shot male	1		[65]
29	Theniet El Had (now a NP)	Winter 1844	Shot, 3 (14 lions in his life)	1	Mokhtar ben el Arbi	[66]
30	Taza vicinity	1844	Lion attack	0		[66]
31	Miliana vicinity	1844	Lions attack on livestock	1	Bugeaud	[67]
32	Gaada (Djebel Amour)	Before 1845	Refuge place	0		[68]
33	Sidi Bel Abbés vicinity	Winter 1845	Shot, large lion	0		[69]
34	Sbeitla, Tunisia	1845	Observations	1	Bruce	[59]
35	Kef vicinity (Tunisia)	Spring 1845	Skin female	0		[70]
36	Mahouna Gorges (near Guelma)	Summer 1845	Shot, 2	1		[71]
37	Bibans	Summer 1845	Roaring	0		[72]
38	Saida	1846		1	Berbrugger 1846	[73]
39	Staouéli	1846	Attack, male	0		[74]
40	Guelma vicinity	Winter 1847	Shot	0		[71]
41	Theniet El Had (actually NP)	Winter 1847	Shot	0		[66]
42	Djebel Guerioun (Aurès Mounts)	Winter 1848	Fresh faeces and an old male seen	0		[71]
43	Montagne des lions (Oran-Arzew)	Winter 1849	Observations	1		[75]
44	Saf Saf valley (Skikda region)	Winter 1849	Observations	1		[75]
45	Zerazer (Aurès Mounts)	Winter 1850	Shot, 2	1		[71]
46	Tebessa	Spring 1850	Fresh male skin	0		[76]
47	Djebel Bouarif (Aurès Mounts)	Summer 1850	Winter refuge; 30 lions in the area	0		[77]
48	Fedjoudj (Aurès Mounts)	1850	Winter refuge	0		[77]

NP = one of several National Parks established across the region in the 1980s–1990s.

**Table 3:** Lion sightings in the eastern Maghreb of Algeria and Tunisia, 1851–1860 (Ksour Mountains, Saharan Atlas, Tell Atlas, Ouled-Nail, Aurès Mountains).

	Location	Season/Year	ObservationType	Solitary(0)/Group (1)	Original source	Ref.
49	Ourten valley Khenchela (Aurès)	Autumn 1851	Observation, male, female	1		[71]
50	Drean	Spring 1852	Frequent, Shot male	1		[78]
51	Baba Ali (Mitidja)	Summer 1852	Observation, male	0		[79]
52	El Diss vicinity	1852	Shot, female	0		[65]
53	Milianah vicinity	Spring 1853	Observation	0		[80]
54	Khenchela (Aurès Mounts)	Summer 1853	Shot, male (Shot 25 lions during his life)	0		[71]
55	Djebel Onk Jemel (Aurès Mounts)	Autumn 1853	Shot, female	0		[71]
56	Ferdjioua vicinity	Spring 1855	SidiBou Akas	1	Vicomte de Noé	[81]
57	Oued Tafna	1855	Tracks	0	Vicomte de Noé	[81]
58	El Harrouch vicinity	1856	Shot, male, female	1		[65]
59	Beni Salah forest	1856–1857	Shot male & female, (shot 39 lions in his life)	1	Ahmed Ben Amar	[82]
60	Medjerda vicinity, Tunisia	1856–1857	Male, cubs, shot female	1	Ahmed Ben Amar	[82]
61	Ain Taoura (vicinity of Souk Ahras)	1856–1857	2 cubs, shot female	1	Ahmed Ben Amar	[82]
62	El Kala vicinity (now a NP)	1856–1857	4 cubs, shot female	1	Ahmed Ben Amar	[82]
63	Ain Temouchent area	Spring 1858	Observation	1		[83]
64	El Kef region, Tunisia	1858	Numerous lions	1		[84]
65	Azzaba vicinity	1858	Shot, 2	1		[83]
66	Skikda-Azzaba	1858	Observation	0		[83]
67	Berrrahal	Winter 1858	Tracks, roaring, 3	1		[85]
68	Oued Saf Saf - Oued Zergua	Winter 1858	Numerous lions	1		[85]
69	Skikda-Azzaba	Autumn 1859	Observation, tracks	1		[86]
70	Boghar forest	1860	Observations	1		[87]
71	Tiaret	1860		1	Leroux	[73]
72	Mascara	1860		1	Leroux	[73]
73	Tazoult-Hammam Essalihine	Winter 1860	Shot male, (Shot 30 lions during his life)	0	Chassaing, F.	[85]

NP = one of several National Parks established across the region in the 1980s–1990s.

**Table 4:** Lion sightings in the eastern Maghreb of Algeria and Tunisia, 1861–1880 (Ksour Mountains, Saharan Atlas, Tell Atlas, Ouled-Nail, Aurès Mountains).

	Location	Season/Year	Observation Type	Solitary (0)/Group (1)	Original source	Ref.
74	Miliana vicinity	Winter 1861	Observations, many lions	1	French man	[88]
75	Djebel Chaambi (now a NP), Tunisia	Spring 1862	Frequent lions	1		[89]
76	Sahel (Algiers vicinity)	Winter 1862	Shot	0	Chassaing, F.	[90]
77	Tebessa mountains, Kasserine, Tunisia	1863	Few lions left on the steppes	1	Citation	[73]
78	Nechmeya vicinity	Autumn 1863	Observation	0		[91]
79	Ourten valley	Autumn 1863	Observations, lion spot	1		[91]
80	Tazoult vicinity	Autumn 1863	Observation, lion killing a cow	1		[91]
81	Mouzaia vicinity (Chréa NP)	1864	Observation, male	0	Le Tell	[92]
82	Tebessa border	1864	Fresh tracks, roaring	0		[91]
83	Beni Salah forest	Spring 1865	Citation	1		[93]
84	Oued Zenati vicinity	Spring1865	Shot	0		[93]
85	Djebel Riless	Summer 1866	Shot, 3	1		[66]
86	Azzaba vicinity	1866	Shot, male, female	1		[65]
87	Djebel Tangout	1866	Observation, male after burning forest	0		[65]
88	Ras El Ma (Azzaba)	1866	Observation, male	0		[65]
89	Oued Saida	1867	Observations	1		[94]
90	Djurdjura (now a NP)	1867	Observations	1		[95]
91	Fetzara lake	1867	Observations	1		[95]
92	Feriana, Tunisia	1868			Monchicourt	[73]
93	Ouarsensis	1869			Leroux	[73]
94	Djebel Bouarif (Aurès Mounts)	1869	Winter refuge, observed and tracks also seen	1		[74]
95	Souk Ahras vicinity	1873	Shot, 5	1		[54]
96	Djebel Bissa	Winter 1874	Observation	0	El Mobacher	[96]
97	Annaba vicinity	Spring 1875	Observations	1		[59]
98	Ain Mimoun (Aurés Mounts)	Spring 1875	Observations, 2 lions roaring	1		[59]
99	Skikda vicinity	1875	Shot 6 lions	0	Cheret, C.	[97]
100	Souk Ahras vicinity	1877	Shot, 3	1		[54]
101	Souk Ahras vicinity	1878	Shot, 4	1		[54]
102	Souk Ahras vicinity	1879	Shot, 5	1		[54]
103	Tebessa forest	1879	Twenty lions killed/year	1		[98]
104	Souk Ahras vicinity	1880	Shot, 3	1		[54]
105	Northwest Tunisia	1880	Four shot, one photographed	1		[99]

NP = one of several National Parks established across the region in the 1980s–1990s.

**Table 5:** Lion sightings in the eastern Maghreb of Algeria and Tunisia, 1881–1900 (Ksour Mountains, Saharan Atlas, Tell Atlas, Ouled-Nail, Aurès Mountains).

	Location	Season/Year	Observation Type	Solitary (0)/Group (1)	Original source	Ref.
106	Djurdjura (now a NP)	1880–95	Shot by French man	0		[52]
107	Aurès Mounts	1880–95	Shot	0		[52]
108	El Kala (vicinity NP)	1880–95	Several shot	1		[52]
109	Souk Ahras vicinity	1881	Shot, 1	0		[54]
110	Ain Drahem, Tunisia	1881	Observation	1		[54]
111	Feidja (now a NP), Tunisia	1881	Observation	1		[54]
112	Khang el Melah (Djelfa)	Summer 1881	Citation	1	Guy de Maupassant	[100]
113	Djebel Chelia (Aurès Mounts)	1884	Last lions in cedar forest	1		[52]
114	Zaccar (Djelfa)	1884	Isolated kills	1		[52]
115	Tebessa	1885	Shot, male	0		[101]
116	Ksenna forest	1886	Observations	1		[102]
117	Ghardimaou, Tunisia	1887	Lions in forest	1	Lataste, 1887	[99]
118	Feriana/Medjerda forest, Tunisia	1887		1	Lataste, 1887	[99]
119	Djebel Mssid vicinity	1887		1	Lataste, 1887	[99]
120	Ghardimaou/Souk Ahras (border area)	1888	Shot, male	0		[103]
121	Seraidji (Edough Mount)	Summer1890	Shot, young female	0		[103]
122	Djedeida, Tunisia	1890	Drowned young lion in dam	0		[52]
123	Babouch (South Tabarka), Tunisia	1891	Shot, male	0		[104]
124	Souk Ahras vicinity	1891	Shot male	0		[104]
125	Edough Mount	1892	Observations, full grown male	0	Werner	[52]
126	Ain Beida	Spring 1893	Shot male	0	Langelet, P.	[105]
127	Batna vicinity	1893	Shot female	0		[104]
128	Oued Sahel valley	1893	Observations	1		[106]
129	Isser Valley	1893	Observations	1		[106]
130	Sebaou valley	1893	Observations	1		[106]
131	Thabourt Bouzgueur	1893	Observations, Winter refuge	1		[106]
132	Aurès Mounts	1894	Lion hunting a horse	0	Germaine Tillon	[107]
133	Djebel Amour	1898	Numerous lions	1		[108]

NP = one of several National Parks established across the region in the 1980s–1990s.

**Table 6:** Last lion sightings recorded in the eastern Maghreb of Algeria, 1900–1960 (Ksour Mountains, Saharan Atlas, Tell Atlas, Ouled-Nail, Aurès Mountains).

	Location	Season/Year	Observation Type	Solitary (0)/Group (1)	Original source	Citation Text S1
134	Beni Salah (Chréa NP vicinity)	1900’s	Observations	1	Inhabitants of Beni Salah	[109]
135	Zaccar-AinTorki	1910–12	Observations	0	MrsDedreuil-Paulet	Difallah, pers. comm.
136	Aurès Mounts	1911	Shot, Male, female	1	Sassorossi family	[107]
137	Bejaia vicinity	1912	Shot	0		[110]
138	Ain Sefra	1912	Shot	0	Khazene, A,	Fellous, pers. comm.
139	Biskra (lion probably from Aurès ranges)	1917	Old lion	0	Seen by the writer	[111]
140	Djebel Tameda, south of Boussemghoun	*c*.1920	Last lion shot in theSaharan Atlas	0	Gueniche Ahmed	Bahmane L to Fellous
141	Between Ain Talawane and Ain Roua (Setif)	Late 1920s	Observation (in spring)	1	Report by old man	Difallah, pers. comm.
142	Guenzet-Babor Mount(North Setif)	1930	Observation	0	Interview local people	[112]
143	North Setif	1930	Observations	1	Interview local people	[112]
144	DjebelDirah (Sour El Ghozlane)	1930s	Shot	0	Kalem, pers. comm.	Fellous
145	Boussam (Menaceur-Zaccar mounts)	Winter 1935	Male lion shotattacking a cow	0	Bounaceur Farid	Fellous
146	Djebel es Somm (Djebel Amour)	1935	Male lion attacked a camel	0	Hamami Bachir	Fellous
147	Unknown location in Algeria	1943	Shot		Keeling, pers. comm.	[113]
148	North Setif	Late 1940s	Observations	1	Interview local people	Difallah, pers. comm.
149	Beni Ourtilane (North of Setif)	1956	Observation	0	People on a bus	[112]

Morocco and the Morocco-Algeria border are considered as the western Maghreb, whilst the eastern Maghreb covers Tunisia and northern Algeria. The meridian bisects sightings west and east, the most proximal being separated by 220 km of desert and temporally by nearly 70 years. The nearest contemporaneous sightings across this divide occurred in 1912 and are separated by a distance of over 450 km. A small central population in the Saharan Atlas (observed in 1898, 1912, 1920 and 1935) possibly traversed both regions but, owing to the size of the dataset, exclusion of these 3 sightings made very little difference.

### 2.2 Inferring Extinction Dates

While the last sighting of a species is often used as the time of extinction, it rarely corresponds to the true extinction date [33].

Following Solow [33], let sighting times in years be order *t*_1_ *<t*_2_ *<…<t_n_*, where *t*_1_ = 0. Sampling from the uniform distribution, the unbiased estimate of extinction time is 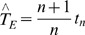
(1) and the expected year of extinction is 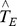 plus the year of *t*_1_. The upper bound, 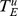, of 1-α CI for *T_E_* is
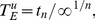
(2)where α = 0.05 (after [33]). Several other methods of inferring dates of extinction have been developed (see Solow [33] for a review). However, in a study of putatively extinct North American birds, Vogel et al. [34] showed that the simple Poisson process model, used here, had as good or better fit than other more complex models, such as the truncated exponential and Weibull.

Here we calculate the extinction dates for sightings of lions in the Western (Table 1; *n* = 16) and Eastern Maghreb (Tables 2–6; *n* = 66).

### 2.3 Testing Behavioral Change

We tested whether lions appear to change their behavior as populations decreased in size to operate singly (‘0′) rather than in groups (‘1′) as a binary set of sightings [35].

In a series of *N* sightings, *X*_1_, *X*_2_,…, *X_N_*, each sighting *X_i_* is coded as *X_i_* = 1 or *X_i_* = 0. Of the *N* sightings, let *m* equal the number of sightings of lions in groups and *n* equal the number of sightings of lions singly. Then
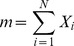
(3)and *n* = *N–m*. The cumulative number of group sightings at each point is then determined. This frequency is
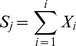
(4)where j = 1,2,…,N.

The statistic for testing the hypothesis of change is
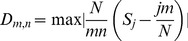
(5)


The expression is evaluated for all values of *j* from 1 to *N*-1. *D_m,n_* is the largest absolute difference observed in the sequence. We may reject the *H*_o_ at 0.05 if 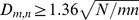


Here we test the null hypothesis that there has been no change in the probability of sightings of lions in groups over time in the Western and Eastern populations.

### 2.4 Estimation of Generations between Moroccan Royal Lions and Wild-caught Specimens

Hemmer [20] suggested that 20 generations separated Moroccan Royal lions surveyed in 1974 from wild lions captured *c.*1899, suggesting a 3.75-year generation time and multiple opportunities for hybridization with non-Barbary lions. The average age at which a wild female lion gives birth is 6.5–8 years [11], [36]. In the Moroccan Royal lion studbook, the mean generation time through to today’s juveniles is 7.4 years (STD±3.2) in a range of 6.6–10 years [37]. The 30 breeding females since 1974 have a mean age at first surviving litter of 6.3 years (STD±2.9). These observations suggest that Moroccan Royal lions have a generation time similar to the birthing age for wild lions, 6.5 years [36]. We calculate the number of generations between the animals moved to Temara Zoo and the likely last wild-caught animals, based on a calculated inferred extinction date. We also calculate the number of generations occurring between sightings of wild Barbary lions as an indicator of persistence.

## Results

### 3.1 Sightings of Lions in North Africa

**Figure 1:**
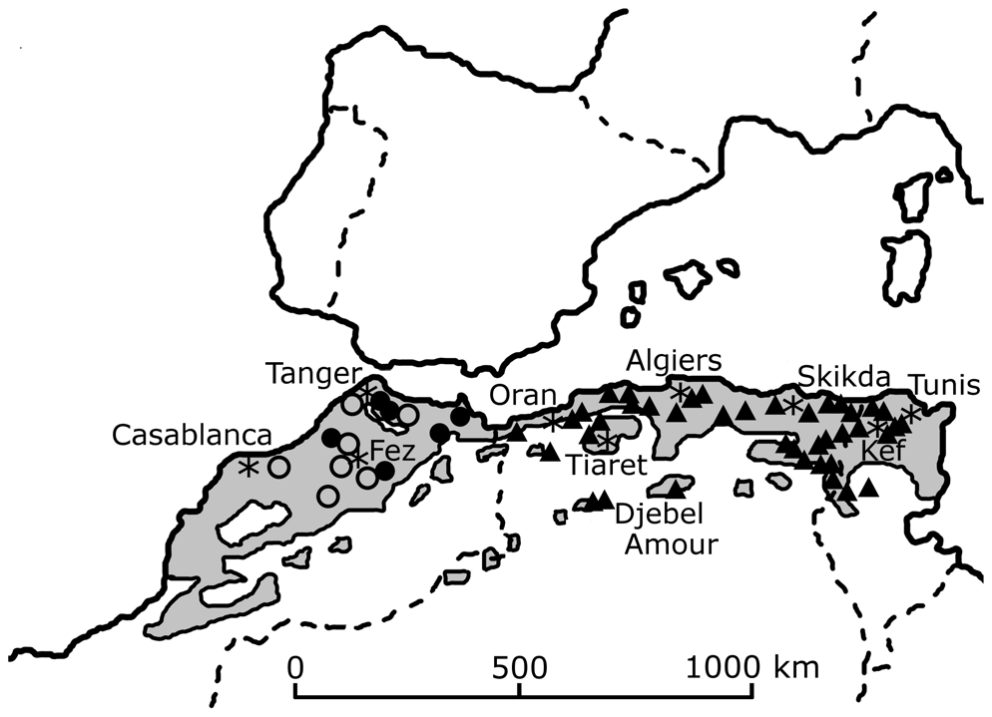
Distribution of historical reports of lions in North Africa (AD 1500–1900). Grey shading indicates Mediterranean scrubland ecosystems [8]. Earliest accounts in the western Maghreb from 16^th^ to the 18^th^ century are indicated as open circles [7], [19]. Documented sightings in known years from 1800 to 1900 are indicated as black circular markers in the western Maghreb (1–7 in Table 1); triangular markers indicate sightings in eastern Maghreb (22–133 in Tables 2–6). Asterisks (*) denote locations of human population centers. Dashed lines indicate national boundaries.

**Figure 2:**
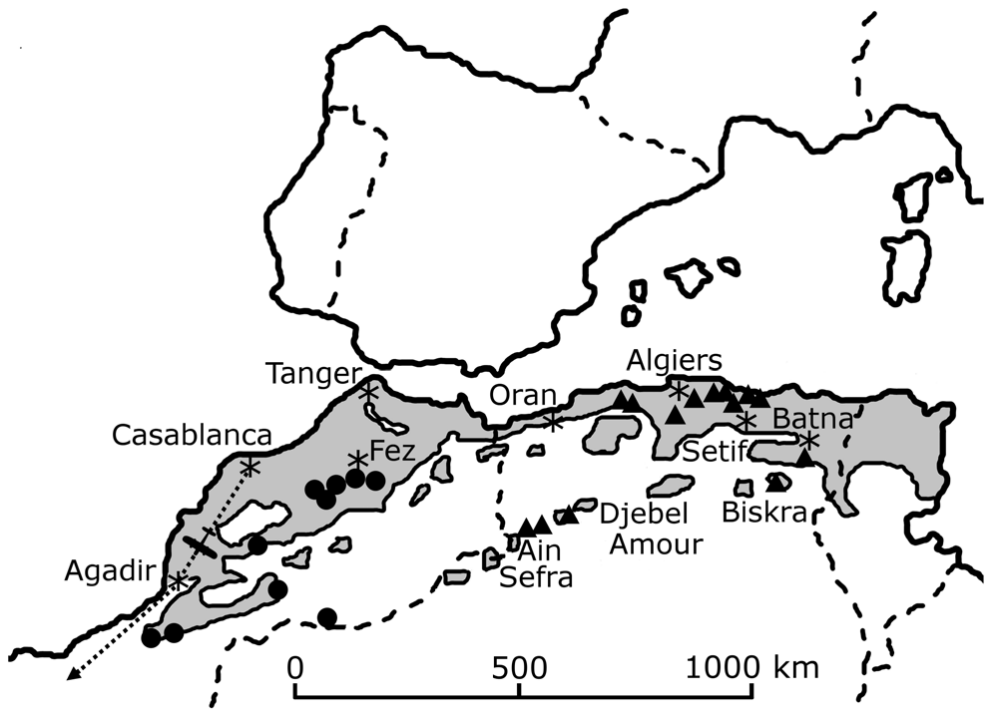
Distribution of recent sightings of lions in North Africa (AD 1900–1960). Grey shading indicates Mediterranean scrubland ecosystems [8]. Circular markers indicate sightings in western Maghreb (8–21 in Table 1); triangular markers indicate sightings in eastern Maghreb (134–149) from incidents described in Table 6. The dotted line indicates the air route across the Atlas Mountains (Casablanca-Agadir-Dakar) during which the last wild lion was photographed. Asterisks (*) denote locations of human population centers. Dashed lines indicate national boundaries.

**Figure 3:**
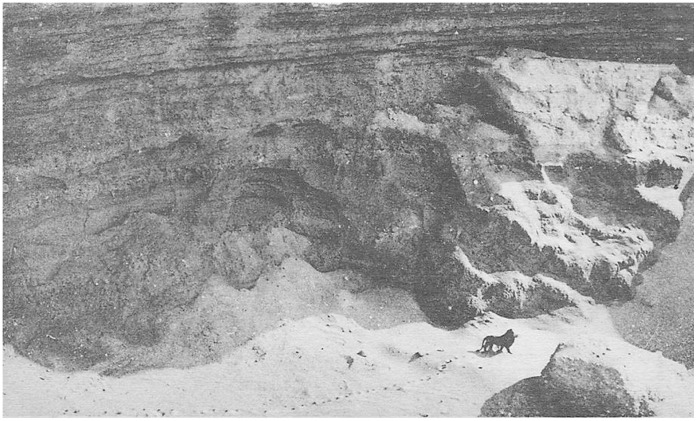
A lion seen in the Atlas Mountains, during a flight on the Casablanca-Dakar air route. The photograph taken by Marcelin Flandrin in 1925 is the last visual record of a wild ‘Barbary’ lion of North Africa.

The list of recent historical lion sightings in North Africa includes data for the western Maghreb from 1840 to the 1940s (Table 1) and the eastern Maghreb from the 1830s to the 1950s (Tables 2–6). Sightings become infrequent in the eastern Maghreb after the 1890s and in the western Maghreb after the 1920s. Our examination of historical accounts reveals that lions occupied the Saharan Atlas (Fig. 1), much further south than previously reported by Schnitzler [27]. The precise location of the aerial photograph on the Casablanca-Dakar route (Fig. 2) is unknown, however our research has identified that it was taken in 1925 when flights commenced [38]. A postcard edition of the image has recently been discovered with the caption “Un lion photographié en avion dans l'Atlas” (Fig. 3). Since this predates discussions on the extermination of lions in the region [19], its significance at that time was then unknown. The photograph by Flandrin is the last known image of a wild Barbary lion.

### 3.2 Recent Extinction of Lions in North Africa

In recent years it has become widely asserted that the animal shot in 1942 on the Tizi-n-Tichka pass in Morocco’s High Atlas Mountains [18] has been considered the last wild Barbary lion [4]. However, our analysis suggests that wild lions actually persisted longer in Algeria until 1958 (*T_E_*), with an upper bound for the 95% CI of 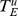 = 1962. This is 10 years after 1948 (*T_E_*), the estimated extinction date of the western (Morocco) population. However, the estimated 95% confidence intervals have a substantial overlap: with upper bounds (*T_E_*) of 1962 for the Algerian population and 1965 for the western (Morocco) population.

### 3.3 Change in Behavior

The frequency of observations, particularly after the 1920s, which involves solitary animals rather than groups (Tables 1–6), may be entirely due to a dwindling population with lower density or a potential change in social behavior (from group to solitary living). Owing to the uncertainty surrounding some sightings, and therefore the order of occurrence of single and group sightings of lions, we analyzed the date at the two extremes; all groups occurring as late as possible and then as early as possible (only relevant to the eastern population). For the western population *D_m,n_* = 0.351 compared with a critical value of 0.658 (*N* = 18), so that we cannot reject the *H*_o_ and suggest that there has been no change in behavior. Likewise for the eastern population no change in behavior was detected even though the inhabited areas are adjacent to human settlements; *D_m,n_* ranged from 0.235 to 0.249 compared to a critical value for both of 0.250 (*N* = 121).

### 3.4 Persistence of Lions

If we consider the gap size between sightings, lions appear able to persist whilst unseen by humans for at least a generation, if not two. A more rigorous analysis of gaps sizes for sightings is not possible due to ambiguity in some sighting dates. In the western Maghreb lions went unseen for 10 years, reducing to 3–5 years towards the end of the record. In the eastern Maghreb the gap sizes between later sightings were generally greater than 5 years, although precision is difficult owing to uncertainty in some dates. In one case the gap could be >10 years (1900s to 1910–1912).

### 3.5 Generations between Moroccan Royal Lions and Wild-caught Specimens

**Figure 4:**
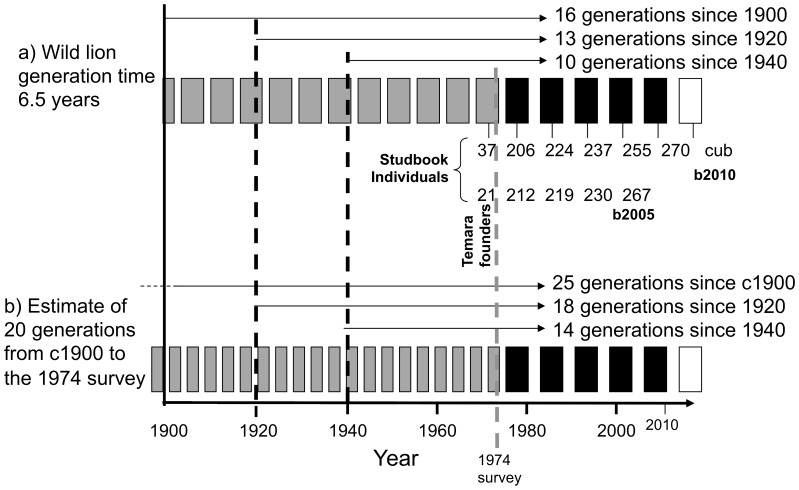
Estimates of captive generations since wild collection in North Africa for current Moroccan Royal lions. Grey boxes indicate estimated lion generations based on suggestions by: (a) Packer et al. [38], and (b) Hemmer [20]. Black boxes are the five known generations in the European studbook [13] since the 1974 survey at Temara Zoo. Generational positions for two studbook maternal lines are illustrated for a female cub (white box) born to studbook female 270, and a young male (267) born to female 230, tracing to founder females 37 and 21 respectively.

Current breeding animals in the Moroccan Royal Lion studbook [13] are separated from the Royal Palace animals by just four generations (in one case, three generations) and recent cubs [39] are five generations removed. Hemmer’s [20] estimate of generations between wild ancestors and the lions in the Royal collection in the 1970s, would suggest that today’s surviving descendants stand 25 generations from wild ancestors (Fig. 4). However, assuming a generation time of 6.5 years [36], our analysis places today’s Moroccan Royal lions as much closer descendants; with the youngest breeders only 16 generations removed from wild ancestors (Fig. 4).

## Discussion

Our analysis suggests that relatively recent sightings of lions (1940s and 1950s) are not exceptional. Whilst we corroborate recognized sightings in Morocco in the 1940s, we also suggest that lions persisted in Algeria into the late 1950s, nearly seventy years later than considered in previous reviews. Barbary lions experienced a combination of factors of decline consistent with the threats to modern-day felids; habitat availability, wild prey availability, livestock husbandry and management, human behaviour, land use and socio-economics [40], [41].

A more recent extinction date for Barbary lions raises several questions: (a) how lions managed to persist in degraded North African ecosystems; (b) whether later persistence in North Africa provides insights for conserving marginal lion populations in West and Central Africa; (c) whether wild Barbary lions were more recently taken into captivity, making current animals closer descendants than previously considered.

### 4.1. Recent Persistence of Lions in North Africa

Previous assumptions that lions have been absent from North African ecosystems for most of the past century is challenged by our analysis. We suggest that small populations persisted largely undetected by humans for several generations, indeed many decades. In Morocco, an inhospitable interior always made encounters with lions rare, whilst in Algeria sightings across the population’s range occurred on an almost annual basis up to 1894, although thereafter less frequently than every 1 lion generation (6–7 years). Our calculation of the time of extinction (*T_E_*) corresponds with the recent suggestion that the last Barbary lion was probably lost in the destruction of forests north of Setif in 1958 during the French-Algerian War [25].

### 4.2 Adaptation of Lions in North African Ecosystems

Up to the late 1800s, hunters reported lions traversing from northwest Algeria, westwards into Morocco and from northeastern Algeria eastwards into Tunisia [19]. After the 1880s, the pattern of sightings suggest that lion populations retreated broadly in two directions; in Morocco southwards away from coastal regions through the Rif, Middle and High Atlas Mountains and the Saharan fringes; and in Algeria eastwards into the Tell Atlas and the Aurès Mountains bordering Tunisia. In both regions small populations survived at low densities in remote areas for several generations. Literature and oral accounts suggest that lions persisted through certain behavioral adaptations (hunting domestic livestock, engaging in nocturnal activity, living in small groups or pairs) and shifts in range (leaving deforested localities, moving to outlying areas and higher altitudes, and following water points in arid regions). Many of these particular behavioral adaptations have since been observed in contemporary populations of lions in human-dominated landscapes in sub-Saharan Africa [42], [43] as well as in restricted available habitat in India [11].

Our analysis of historical records in the Maghreb suggests that lions appear not to adapt their social behavior as a response to human habitat pressure. There was no significant change from group to solitary living by lions in the remote western Maghreb regions, whilst importantly even the eastern Maghreb lion populations did not exhibit a significant change in group living in the final decades of their existence, despite increasing encroachment by human populations. Although lion population density is typically lower in desert and semi-desert areas, pride size generally remains comparable in desert ecosystems and moister ecosystems with higher lion density [22]. Interestingly, human presence has been shown to provide advantages to lion cub survival by removing secondary predators [44], and this may have enabled lion persistence in the Maghreb.

Final extirpation of lions through hunting was a response to livestock predation; a prey dependency probably driven by reduced habitat and fewer wild ungulates [18]. This final collapse may have been exacerbated by the species’ behavioral need to remain in social groups with proportionally greater local resource requirements. Group living has been shown to be similarly maintained by lion populations in semi-arid environments such as the Kalahari and Etosha [45] [46]. In contrast, leopards (*Panthera pardus*) still persist at lower population densities in Morocco [1].

### 4.3 Heritage of Moroccan Royal Lions

Moroccan Royal Lions have been described as “an obvious relic of the original Barbary lion gene pool” [20]. Our analysis suggests fewer post-wild generations have occurred during the period where this population has existed solely in captivity. Today’s lions, descended from the Moroccan Royal collection, could be closer relatives to wild ancestors than previously considered. In the absence of definitive nuclear DNA profiles from wild Barbary lion specimens, the precautionary principle suggests that Moroccan Royal lions should be conserved as descendants of the Barbary lion until science can tell us otherwise. The Moroccan Royal lions offer one of the few scenarios in which restoration of lions into regions where the species is long extinct could be envisioned as having useful conservation value [47].

### Conclusions

Insights from historical sightings are relevant to current lion conservation. We suggest that wild lions persisted in the Maghreb into the 1950s, much later than previously recognized. The lion is a well-known, visible and potentially threatening species, yet small populations survived in North Africa decades after being generally considered extinct. This persistence reflects the recent rediscovery of a small population of Barbary leopard nearly 20 years after the last previous sighting and a decade since being declared extinct [1] [48]. Careful consideration should be given to mammalian carnivores currently presumed extinct or near-extinct in other regions, coupled with a greater understanding of extinction patterns and the conservation potential in relict populations.

Finally, we suggest caution when considering the current conservation status of lions. Although lions in the Maghreb adapted to reduced population density, prey availability and habitat encroachment, our analysis reveals that lion group-living behavior did not change significantly as human pressures increased. As a pride-forming species [45], *P. leo* populations are prone to collapse, whereas other felids may survive at lower local population densities by not living in social groups [49]. Lions in today’s small populations in Central and West Africa persist [16] [50], even if rarely seen, in fragmented remnants, yet clearly exist at the edge of a precipitous drop into extinction. Continued, carefully considered conservation effort remains vitally important.
